# DNA-based identification reveals illegal trade of threatened shark species in a global elasmobranch conservation hotspot

**DOI:** 10.1038/s41598-018-21683-5

**Published:** 2018-02-20

**Authors:** Leonardo Manir Feitosa, Ana Paula Barbosa Martins, Tommaso Giarrizzo, Wagner Macedo, Iann Leonardo Monteiro, Romário Gemaque, Jorge Luiz Silva Nunes, Fernanda Gomes, Horácio Schneider, Iracilda Sampaio, Rosália Souza, João Bráullio Sales, Luís Fernando Rodrigues-Filho, Lígia Tchaicka, Luís Fernando Carvalho-Costa

**Affiliations:** 1 Universidade Federal de Pernambuco, Programa de Pós-Graduação em Biologia Animal, Departamento de Biologia, Cidade Universitária, CEP 50670-901, Recife, Pernambuco Brazil; 2 Centre for Sustainable Tropical Fisheries and Aquaculture, College of Science and Engineering, James Cook University, Townsville, Qld 4811 Australia; 3 Australian Institute of Marine Science, Townsville, Qld 4810 Australia; 4 Laboratório de Biologia Pesqueira – Manejo de Recursos Aquáticos, Universidade Federal do Pará, Campus do Guamá, Rua Augusto Corrêa, 1, CEP 66075-110, Belém, Pará Brazil; 5 Programa de Pós-Graduação em Recursos Aquáticos e Pesca, Laboratório de Biodiversidade Molecular, Universidade Estadual do Maranhão, Cidade Universitária Paulo VI, s/n – Tirirical, CEP 65055-970, São Luís, MA Brazil; 6 Estácio, Faculdade de Castanhal (FCAT), Curso de Licenciatura em Ciências Biológicas, Rodovia BR-316, s/n – Km 60, Castanhal/PA, CEP 68740-420, Castanhal, Pará Brazil; 7 Laboratório de Organismos Aquáticos, Departamento de Oceanografia e Limnologia, Universidade Federal do Maranhão, Avenida dos Portugueses, 1966, CEP 65080-805, São Luís, Maranhão Brazil; 8 Laboratório de Filogenômica, Universidade Federal do Pará, Campus de Bragança, Alameda Leandro Ribeiro – Aldeia, CEP 68600-000, Bragança, Pará Brazil; 9 Universidade Federal Rural da Amazônia (UFRA), Av. Perimetral, 2501, CEP 66077-830, Belém, Pará Brazil; 10 Universidade Federal do Pará, Campus Universitário do Marajó-Breves, Faculdade de Ciências Naturais (FACIN), Avenida Anajás - s/n sl 4, CEP 68800-000, Breves, Pará Brazil; 11 Laboratório de Ictiologia Integrada, Centro de Estudos Avançados da Biodiversidade (CEABIO), Instituto de Ciências Biológicas. Universidade Federal do Pará (UFPA), Av. Augusto Correia, 01, Guamá. CEP 66075-110, Belém, Pará Brazil; 12 Universidade Federal Rural da Amazônia (UFRA), Campus Universitário de Capanema, Rua João Pessoa, N° 121, Bairro Centro, CEP 68700-030, Capanema, Pará Brazil; 13 Laboratório de Genética e Biologia Molecular, Departamento de Biologia, Universidade Federal do Maranhão, Avenida dos Portugueses, 1966, CEP 65080-805, São Luís, Maranhão Brazil

## Abstract

Here, we report trading of endangered shark species in a world hotspot for elasmobranch conservation in Brazil. Data on shark fisheries are scarce in Brazil, although the northern and northeastern regions have the highest indices of shark bycatch. Harvest is made primarily with processed carcasses lacking head and fins, which hampers reliable species identification and law enforcement on illegal catches. We used partial sequences of two mitochondrial genes (COI and/or NADH2) to identify 17 shark species from 427 samples being harvested and marketed on the northern coast of Brazil. Nine species (53%) are listed under some extinction threat category according to Brazilian law and international authorities (IUCN – International Union for Conservation of Nature; CITES – Convention on International Trade of Endangered Species of Wild Fauna and Flora). The number increases to 13 (76%) if we also consider the Near Threatened category. Hammerhead sharks are under threat worldwide, and composed 18.7% of samples, with *Sphyrna mokarran* being the fourth most common species among samples. As illegal trade of threatened shark species is a worldwide conservation problem, molecular identification of processed meat or specimens lacking diagnostic body parts is a highly effective tool for species identification and law enforcement.

## Introduction

Overfishing has a profound impact on elasmobranchs due to the typical life history traits of most target species, including long lifespan, late sexual maturity, low fecundity, and low natural mortality rates, leading to severe population declines^1^. Given this, elasmobranchs are considered the most threatened group of marine fishes in the world^2^.

Fisheries targeting cartilaginous fishes have earned hundreds of millions of dollars since 2000^3^, with an estimated 759,495 tons landed worldwide between 2009 and 2013 alone^4^. The international trade of shark fins has been the primary cause of the recent increase in the overfishing of elasmobranchs^5,6^. Shark fin is a delicacy in many Asian countries, particularly in China, where its market price could reach US$ 1,000 per kilogram^7^. The demand for fins in Asia led to the overfishing of several shark species worldwide^3^, but public pressure and environmental legislation in many countries have been slowly modifying market trends. As a result, there was a decline of up to 40% of the market value of shark fin sales^8^, decreasing the demand for shark fins worldwide^9^.

Despite this change, South Atlantic shark populations are still under intense fishing pressure^10^, given that market demands have now shifted from fins to meat. In Brazil alone, 200,000 tons of cartilaginous fish (unspecified category of sharks, rays and chimaeras) were landed between 2000 and 2011^11^. Brazil is now among the top elasmobranch fishing nations, and figures as possibly the largest shark meat importer of the world^5,11,12^. Nevertheless, official statistics on cartilaginous fish catches are derived primarily from data provided by industrial fisheries.

However, almost half of all Brazilian fishery catches are derived from artisanal operations (small-medium sized vessels of reduced storage capacity), which are typically equipped with unsophisticated gear and practice predatory fishing techniques^9^. The impact of artisanal fisheries tends to be overlooked in fishery statistics due to the difficulty in obtaining reliable catch data, combined with the belief that these operations have a low impact on fishery stocks^13,14^. The inclusion of these data source would increase the official catches by up to 50%^13^, especially in developing countries such as Brazil^11^, where artisanal fishing fleets are large, the regulation agencies are inefficient and poorly equipped for law enforcement activities, and fisheries data are inadequate or imprecise^4^. Most of the catch produced by artisanal fisheries is consumed locally^15^, although the more valuable species and byproducts are typically marketed in major cities or even exported.

In 2014, the Brazil’s Ministry of Environment (MMA) published the ordinance 445/2014 that prohibits the harvesting of certain fish species, including some elasmobranchs. A national plan of action for the conservation of sharks and rays (known as PAN-*Tubarões*) was also developed^16^. Currently, 33% of Brazil’s shark fauna is considered to be in some extinction threat category based on national and international red lists of endangered species^11^. Even so, harvesting and trade of many shark species contemplated by these initiatives has continued, particularly on the North Coast, which along with northeastern Brazil are responsible for the highest rates of elasmobranch bycatch in the country^17^.

Brazil’s North Coast (BNC) harbors the largest continuous mangrove forest in the world, sustaining a major fishing ground. According to Dulvy *et al*.^18^ irreplaceability score, it is one of the world’s elasmobranch conservation hotspots. Nineteen shark species are known to occur in the area, of which 11 (*Ginglymostoma cirratum* Bonaterre 1788, *Mustelus canis* Mitchell 1815, *Carcharhinus porosus* Ranzani 1839, *Carcharhinus perezi* Poey 1876, *Carcharhinus obscurus* LeSueur 1818, *Carcharhinus plumbeus* Nardo 1827, *Isogomphodon oxyrhynchus* Müller & Henle 1839, *Sphyrna mokarran* Rüppel 1837, *Sphyrna tudes* Valenciennes 1822, *Sphyrna lewini* Griffith & Smith 1834, and *Sphyrna tiburo* Linnaeus 1758) are listed in ordinance 445/2014. Under this legislation, harvesting and trade of all these species are restricted, although taxa listed as vulnerable can be fished with caveats. Despite the fact that, under the Brazilian law, sharks must be landed with their fins attached to the body, finned and headless specimens are landed frequently at fishery ports, impeding their accurate morphological identification, and law enforcement^19^.

Molecular identification has already been used to identify misidentified processed shark species affected by fisheries in many countries^20–25^, including Brazil^26–32^. Most studies use a single molecular marker to identify species, but the use of multiple markers can provide more robust results, even complementing the traditional morphological identification^33^. However, no such study has focused on such a large area as the Brazilian North Coast, nor exclusively on sharks. The composition of species exploited by the local shark fisheries is still largely unknown, but these data are necessary to promote sustainable fisheries and conservation measures. In this context, we used DNA-based species identification to investigate the species composition of the sharks landed and traded on the North Coast of Brazil to evaluate fisheries and commercialization of threatened species.

## Results

### Species identification

Overall, 427 samples were identified, revealing the presence of 17 shark species, representing five families (Carcharhinidae, Ginglymostomatidae, Sphyrnidae, Squalidae, and Triakidae) and three orders (Carcharhiniformes, Orectolobiformes, Squaliformes). Most (260) of these samples were identified using COI, and the remaining 167 based on the NADH2 sequences.

*Rhizoprionodon porosus* and *Carcharhinus acronotus* were the most abundant species, contributing 33.10% and 15.88% of all samples, respectively (Table 1). Nine species are included at least in one of the categories of extinction threat (Table 1). None of the species was recorded at all localities, and only one (*R*. *lalandii*) was landed in a unique site (Raposa). Twelve species were identified among samples from Bragança, eleven from Tutoia, nine in both Belém and Raposa, six from Amapá state’s coast, three from Carutapera, and two from Vigia. One sample from Tutoia could not be identified to the species level because of a 98% similarity to both *Squalus brevirostris* and *Squalus megalops* on NADH2. However, whatever the species, this is the first record of *Squalus* from Brazil’s North Coast.

**Table 1:** Shark species identified using COI and NADH2 sequences, their conservation status according to national and international listings, and percentage similarity with sequences deposited in NCBI and BOLD. Threat categories for IUCN and Ordinance 445: DD – Data Deficient, LC – Least Concern, VU – Vulnerable, NT – Near Threatened, EN – Endangered, CR – Critically Endangered, − = no classification. Species are ordered by their frequency of occurrence.

Species	Conservation status (or listing) according to:			COI and/orNADH2	BLAST %	
	Ordinance 445	CITES	IUCN	Number of specimens identified (% of the total)	COI	NADH2
*Rhizoprionodon porosus*	—	—	LC	142 (33.1)	100	100
*Carcharhinus acronotus*	—	—	NT	68 (15.88)	100	100
*Carcharhinus porosus*	CR	—	DD	42 (9.81)	100	100
*Sphyrna mokarran*	EN	Appendix II	EN	40 (9.34)	100	100
*Sphyrna lewini*	CR	Appendix II	EN	18 (4.2)	100	100
*Carcharhinus leucas*	—	—	NT	17 (3.97)	100	100
*Ginglymostoma cirratum*	VU	—	DD	14 (3.27)	100	100
*Isogomphodon oxyrhynchus*	CR	—	CR	14 (3.27)	100	—
*Sphyrna tiburo*	CR	—	LC	12 (2.8)	—	100
*Galeocerdo cuvier*	—	—	NT	12 (2.8)	100	100
*Carcharhinus falciformis*	—	Appendix II	NT	11 (2.57)	—	99
*Sphyrna tudes*	CR	—	VU	10 (2.33)	100	100
*Carcharhinus limbatus*	—	—	NT	9 (2.10)	100	100
*Mustelus higmani*	—	—	LC	8 (1.86)	—	99
*Mustelus canis*	EN	—	NT	8 (1.86)	—	99
*Squalus brevirostris/megalops*	—	—	DD/DD	1 (0.23)	—	98
*Rhizoprionodon lalandii*	—	—	DD	1 (0.23)	—	100
Total				427		

### NADH Dehydrogenase Subunit 2 (NADH2)

Partial NADH2 gene sequences (431 base pairs, bp) allowed the identification of three orders (Carcharhiniformes, Orectolobiformes, and Squaliformes), five families (Carcharhinidae, Ginglymostomatidae, Sphyrnidae, Squalidae, and Triakidae), seven genera (*Carcharhinus*, *Galeocerdo*, *Ginglymostoma Mustelus Rhizoprionodon*, *Sphyrna*, and *Squalus*), and 15 species (Table 2). Average interspecific K2P divergence was 11.3%, with pairwise differences ranging from 3.7% for the two *Rhizoprionodon* species to 23.2% between *Squalus* sp. and *G*. *cirratum* (Table 2).

**Table 2:** Pairwise K2P distances between shark species from Brazil´s North Coast based on NADH2 sequences. Scientific names are abbreviated as follows: *Carcharhinus leucas* (Cleu), *Rhizoprionodon porosus* (Rpor), *R*. *lalandii* (Rlal), *C*. *falciformis* (Cfal), *C*. *acronotus* (Cacr), *C*. *limbatus* (Clim), *C*. *porosus* (Cpor), *Sphyrna lewini* (Slew), *S*. *mokarran* (Smok), *S*. *tiburo* (Stib), *S*. *tudes* (Stud), *Galeocerdo cuvier* (Gcuv), *Mustelus higmani* (Mhig), *M*. *canis* (Mcan), *Ginglymostoma cirratum* (Gcir).

Species	Cleu	Rpor	Rlal	Cfal	Cacr	Clim	Cpor	Slew	Smok	Stib	Stud	Gcuv	Mhig	Mcan	Gcir
Rpor	0.114														
Rlal	0.131	0.037													
Cfal	0.084	0.116	0.120												
Cacr	0.066	0.123	0.131	0.077											
Clim	0.075	0.111	0.115	0.077	0.073										
Cpor	0.097	0.127	0.130	0.092	0.092	0.092									
Slew	0.113	0.106	0.133	0.127	0.115	0.127	0.139								
Smok	0.115	0.112	0.125	0.143	0.129	0.109	0.150	0.098							
Stib	0.112	0.100	0.115	0.132	0.112	0.105	0.131	0.086	0.099						
Stud	0.131	0.116	0.136	0.140	0.129	0.115	0.137	0.095	0.107	0.052					
Gcuv	0.126	0.129	0.143	0.116	0.141	0.130	0.153	0.145	0.143	0.160	0.165				
Mhig	0.159	0.168	0.170	0.150	0.170	0.148	0.167	0.163	0.164	0.172	0.166	0.146			
Mcan	0.150	0.167	0.164	0.152	0.160	0.140	0.147	0.169	0.162	0.170	0.164	0.144	0.047		
Gcir	0.190	0.171	0.174	0.190	0.192	0.190	0.190	0.201	0.184	0.205	0.206	0.195	0.224	0.232	
*Squalus*	0.208	0.185	0.176	0.190	0.190	0.206	0.202	0.204	0.188	0.205	0.199	0.204	0.213	0.214	0.210

### Cytochrome Oxidase Subunit 1 (COI)

COI partial sequences (515 bp) enabled the identification of two orders (Carcharhiniformes and Orectolobiformes), three families (Carcharhinidae, Sphyrnidae, Ginglymostomatidae), six genera (*Carcharhinus*, *Galeocerdo*, *Ginglymostoma*, *Isogomphodon*, *Rhizoprionodon*, and *Sphyrna*) and 11 species (Table 3). Average interspecific K2P distance was 7.5% (Table 3). The lowest distances were recorded between species of the *Carcharhinus* genus, and the highest between Carcharhiniformes and *Ginglymostoma cirratum*. Pairwise distances ranged from 3.3% for *Isogomphodon oxyrhynchus* and *Carcharhinus acronotus* to 18.6% for *G*. *cirratum* and *Sphyrna mokarran*. In all cases, pairwise distances were above the 2% threshold for species delimitation using COI^34^.

**Table 3:** Pairwise K2P distances between shark species from Brazil´s North Coast based on COI sequences. The abbreviations of the species names are as in Table 2, except for *Isogomphodon oxyrhynchus* (Ioxy).

Species	Stud	Smok	Gcuv	Rpor	Gcir	Slew	Cacr	Cpor	Ioxy	Clim
Smok	0.117									
Gcuv	0.116	0.095								
Rpor	0.118	0.095	0.088							
Gcir	0.178	0.186	0.170	0.165						
Slew	0.098	0.100	0.124	0.116	0.174					
Cacr	0.105	0.076	0.077	0.083	0.182	0.092				
Cpor	0.112	0.098	0.084	0.086	0.181	0.105	0.045			
Ioxy	0.105	0.092	0.075	0.085	0.180	0.105	0.033	0.039		
Clim	0.107	0.096	0.077	0.089	0.168	0.111	0.051	0.056	0.057	
Cleu	0.107	0.094	0.091	0.089	0.176	0.107	0.043	0.045	0.053	0.047

## Discussion

Given the lack of morphological traits for species identification of processed shark samples, we used molecular markers to assess the species composition of the sharks landed and traded on Brazil’s North Coast. DNA-based identification revealed seventeen species, nine (53%) of which are listed in some category of threat (IUCN, CITES or Ordinance 445). If the species under the Near Threatened category are also considered, the number increases to 13 (76%). We also provide the first record of the family Squalidae for the BNC. The present study is so far the largest one focusing exclusively on the shark trade monitoring using molecular techniques in Brazil.

The trade of endangered shark species is a worldwide phenomenon^20,23,35^. Results herein were not surprising, given Brazil’s status as one of the seven leading producers and consumers of shark meat^2,4,5^, and the number of vulnerable or endangered endemic species found in the country’s coast^13,19^, which now corresponds to 33%^11^. However, the presence of so many species at risk of extinction in one of the most important fishing zones and elasmobranch conservation hotspots is a major concern both for conservation and the long-term sustainability of local fisheries. In Guyana’s coast, the western part of the elasmobranch conservation hotspot where BNC is classified^18^, thirteen shark species were identified using COI^36^, of which nine (~70%) are listed as either threatened, vulnerable or near threatened by IUCN. Only four species are not shared (*Isogomphodon oxyrhynchus*, *Mustelus canis*, *M*. *higmani*, *Squalus* sp.) between our study, and both reinforce the ineffectiveness of the official fisheries statistics and regulations in most of the conservation hotspot area.

A greater diversity of shark species has been identified being harvested in other parts of the world^23,35,37,38^. However, there is considerable overlap among studies involving only species with a global distribution, such as *Galeocerdo cuvier*, *Carcharhinus limbatus*¸ *C*. *leucas*, *C*. *falciformis*, and *Sphyrna lewini*. Here, we highlight the unique diversity of the shark fauna of Brazil’s North Coast, in particular, species with a restricted geographic distribution, such as *Ginglymostoma cirratum*, *Mustelus canis*, *M*. *higmani*, *Isogomphodon oxyrhynchus*, *C*. *porosus*, *Rhizoprionodon porosus*, *R*. *lalandii*, *S*. *tiburo*, and *S*. *tudes*. This reinforces the importance of the region for global shark conservation purposes, as emphasized by Dulvy *et al*.^12,18^ and Davidson & Dulvy^2^.

Maranhão state’s coast is not included in the hotspot zone. Nevertheless, here we show a significant number of threatened species and specimens being landed and commercialized in the state, mainly in Tutoia (area of the Parnaíba river delta). Thus, we recommend that the 640 km coastline of Maranhão should be included in the hotspot 3 proposed by Dulvy *et al*.^18^ as part of this major elasmobranch conservation area.

We provide evidence of an ongoing trade of threatened shark species on Brazil’s North Coast, thus indicating the practice of environmental crimes by local fisheries. Previous studies using molecular data show that the Brazilian shark trade involves a smaller diversity than that recorded here, including endangered species such as *Sphyrna lewini*^26,30–32,39,40^. Based on these findings, we reinforce the need to use molecular identification tools for monitoring shark fisheries and law enforcement, especially when accurate morphological identification is difficult or even impossible.

Forensic genetic studies have been contributing to the more effective monitoring of the fish trade worldwide^20,37^. Most are based on Hebert *et al*.^34^ proposal of using a single molecular marker (COI) for animal species identification, among other reasons, due to the ongoing decline in the numbers of taxonomists worldwide. In fact, COI is an effective tool for shark species identification^41^, because, as in the case of the BNC sharks, it exhibits interspecific genetic distances above the 2% threshold used as criterion for species delimitation. However, other markers that evolve at different rates may be important to resolve some taxonomic uncertainties that COI is unable to deal with^37,42^, as we show here for NADH2-based species identification.

**Figure 1:**
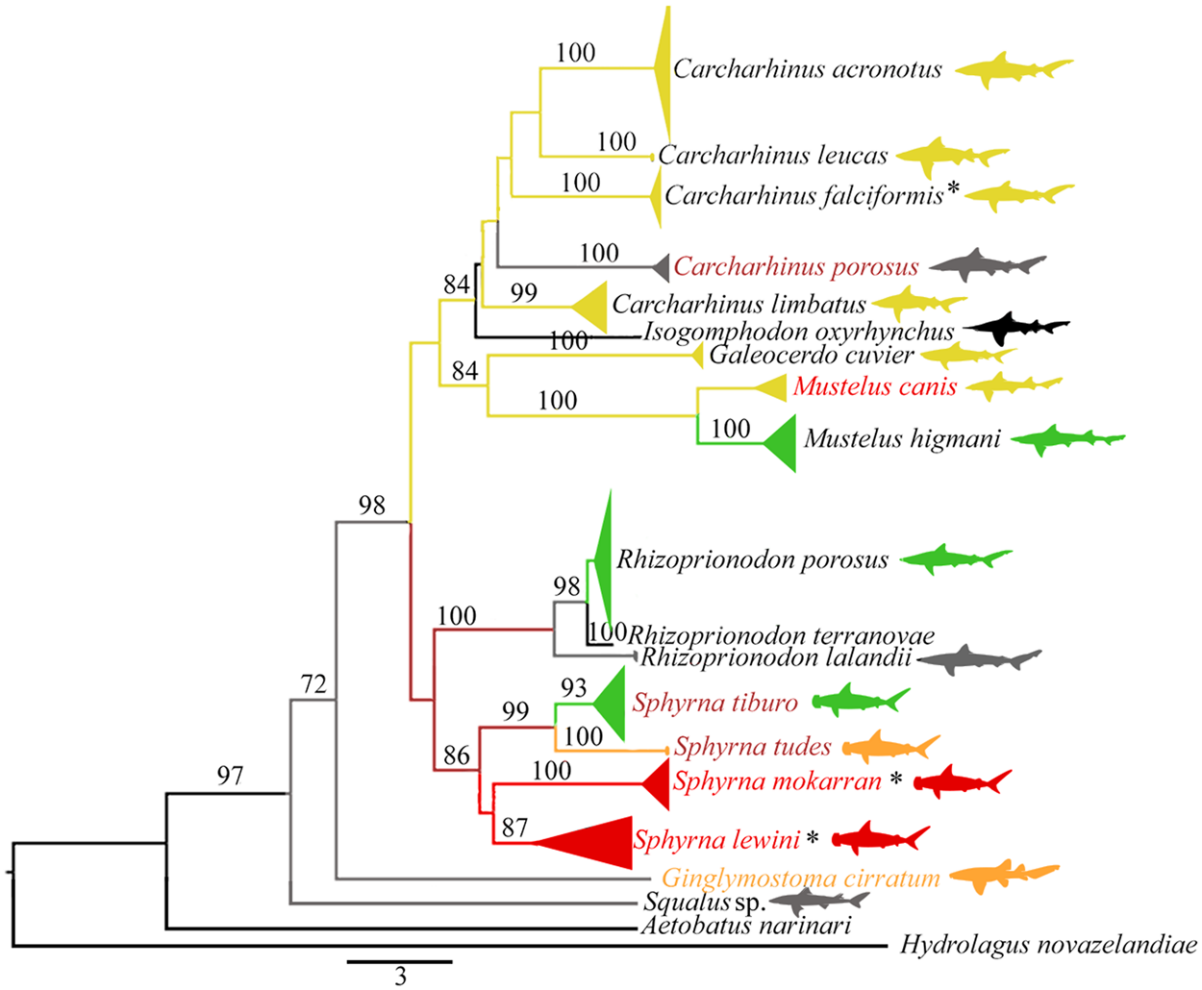
Neighbor-Joining tree for the NADH2 sequences of sharks from Brazil’s North Coast. Sequences of *Isogomphodon oxyrhynchus*, *R*. *terranovae*, *Aetobatus narinari*, and *Hydrolagus novazelandiae* were obtained from NCBI – black branches. Bootstrap values below 70 are not shown. Branch colors follow IUCN extinction threat categories in Table 1 (dark red = CR, red = EN, orange = VU, yellow = NT, green = LC, gray = DD). Species with colored names are listed in Ordinance 445/2014. Asterisks correspond to species listed in CITES Appendix II.

**Figure 2:**
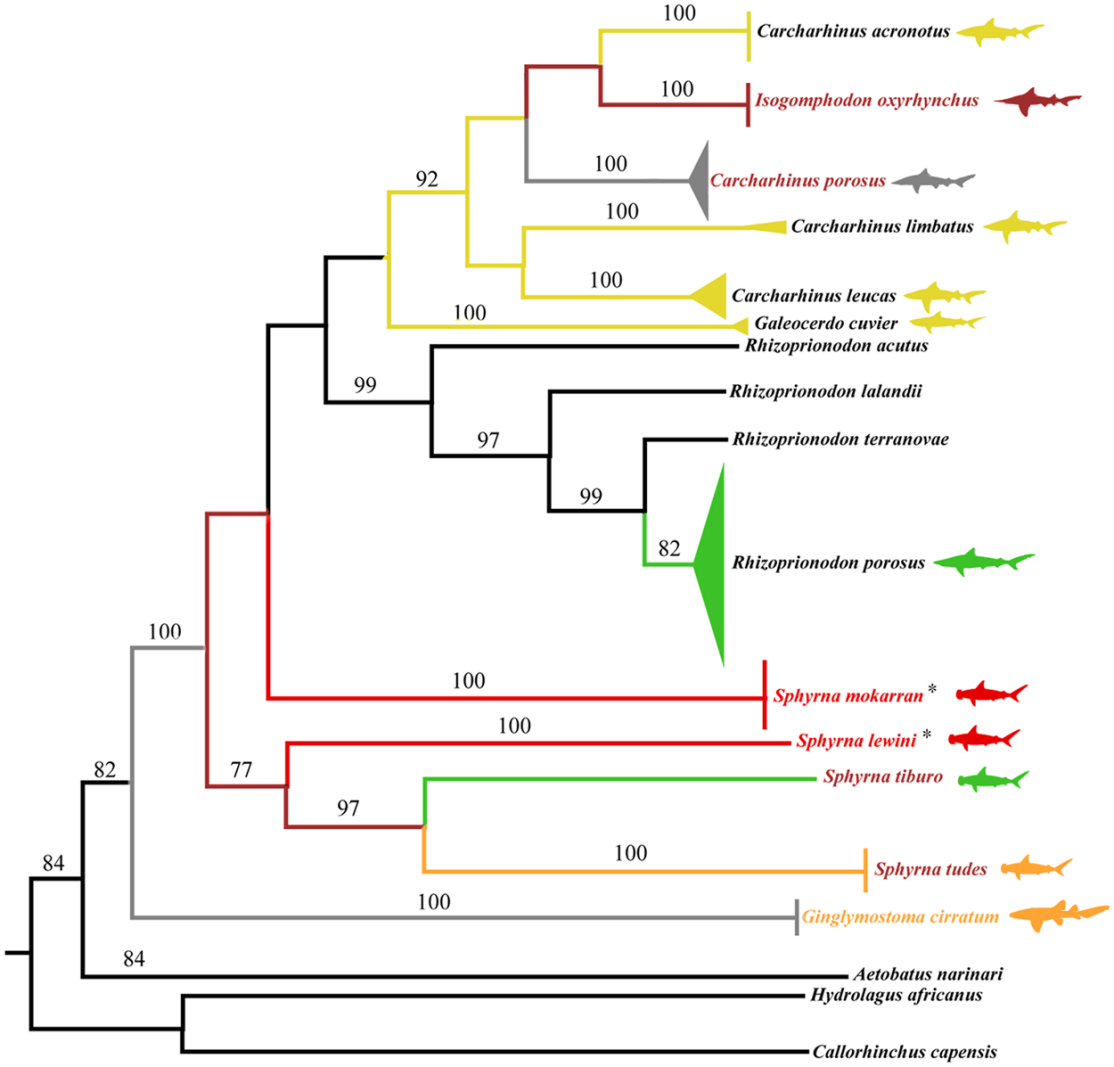
Neighbor-Joining tree for COI sequences of sharks from Brazil’s North Coast. Sequences of *Rhizoprionodon terranovae*, *R*. *lalandii*, *R*. *acutus*, *Aetobatus narinari*, *Hydrolagus africanus* and *Callorhinchus capensis* were obtained from NCBI - black branches. Bootstrap values below 70 are not shown. Branch colors follow IUCN extinction threat categories in Table 1 (dark red = CR, red = EN, orange = VU, yellow = NT, green = LC, gray = DD). Species with colored names are listed in Ordinance 445/2014. Asterisks correspond to species listed in CITES Appendix II.

The NADH2 marker has also been used to identify elasmobranch species^37^. It was decisive for the identification of some species in our samples, such as *Mustelus higmani* which does not have COI sequences deposited in any public database. Furthermore, NADH2 was more effective to identify *Rhizoprionodon* species, because COI sequences had 100% match with both *R*. *porosus* and *R*. *terranovae*, while NADH2 had 100% match with *R*. *porosus* and 97% with *R*. *terranovae*. Despite this, both markers produced reliable species delimitation in the NJ trees (Figs 1 and 2). That raises the suspicion that some sequences from this genus deposited in the public databases might belong to misidentified specimens, as suggested by Kolmann *et al*.^36^, which reinforces the importance of using multiple markers for species identification.

Studies carried out in the BNC between the 1980s and the late 2000s demonstrate that *C*. *porosus* used to be the most commonly harvested species^26,43^. Furthermore, *Sphyrna tiburo*, *R*. *porosus*, *R*. *lalandii*, *S*. *lewini*, and *Isogomphodon oxyrhynchus* used to be the following most abundant elasmobranch fishing resources in Maranhão state, in that order^43^. Since then, *C*. *porosus*, *S*. *tiburo*, *S*. *lewini*, and *I*. *oxyrhynchus* have been included in Brazil’s endangered species list due to severe population decline throughout their ranges. The most evident case is of *I*. *oxyrhynchus*, which is considered to be on the brink of extinction due to overfishing^44^.

Some studies reveal the impact that fisheries have in changing species abundances in other regions of the globe^45,46^. Thus, we argue that a shift in species abundance has occurred in the BNC, based on our results, the scientific studies carried out in the BNC in the last 30 years, and the current species conservation statuses. *R*. *porosus* is now the most abundant species, while the once previously most common one (*C*. *porosus*) has suffered a considerable catch reduction, possibly due to population declines. Nevertheless, further fisheries independent studies are required to test if this pattern reflects changes in the species abundance in the assemblage and if the major cause for these declines is indeed overfishing.

As the most species rich family among sharks, the requiem sharks (family Carcharhinidae) correspond to the majority of the shark trade worldwide^20^ and are considered a priority group for conservation due to its common harvest in unregulated and unreported fisheries^18^. Carcharhinidae species compiled the vast majority of the samples identified here (418 specimens, ~74%), but neither *R*. *porosus* (33.1%) nor *C*. *acronotus* (15.88%), the most abundant species, are threatened with extinction. Nevertheless, *C*. *acronotus* is classified as Near Threatened (Table 1), and its populations are known to be declining worldwide^47^. A three-fold smaller proportion (5.8% of the identified samples) of *C*. *acronotus* is reported in Guyana’s coast^36^. This species reproduces biennially with females reaching sexual maturity at around 4.5 years, and a mean of 3.5 offspring per gestation^48^, resulting in low rates of intrinsic population growth^49^. Taking these into account, the relatively large number of *C*. *acronotus* specimens (n = 68) identified in the BNC may be misleading. Further population analyses, including assessments of intra-population genetic diversity, are important to establish its current conservation status and to develop effective conservation measures for the species at the BNC.

The relatively high abundance of *R*. *porosus* may reflect its more favorable life history traits, especially its continuous reproduction^49,50^, which may guarantee a recruitment rate higher than fishing pressure. Nonetheless, there are no demographic data on the species in the area, which impedes a reliable assessment of the impacts of an increasing fishing pressure on its populations. In Guyana’s coast, *Rhizoprionodon* species were also the most abundant sharks caught by artisanal fisheries^36^. However, *R*. *lalandii* (18.9%) was the most common species, while *R*. *porosus* composed only 3.8% of the samples. Differently, we found an extremely low number of *R*. *lalandii* specimens in the BNC samples (0.23%), which is puzzling considering that *R*. *porosus* and *R*. *lalandii* were both abundant in Maranhão state during the 1990s^51^. There is no reason to assume that fishing gear used by local fleets would target *R*. *porosus* selectively over *R*. *lalandii*. This is the first study to confirm the occurrence of *R*. *lalandii* in the BNC based on genetic evidence, but there are no data on the local habitat use or migration patterns of either species. This reinforces the need of fine scale population data for a better assessment of the conservation status of *R*. *lalandii*.

The smalltail shark, *Carcharhinus porosus*, was the third most abundant species in BNC (9.81%), although it was rarer in comparison with other studies in both Maranhão state and Guyana’s coast^36,43^. In fact, it was the most abundant shark species harvested in the area during the 1990s, representing 43% of the total catch, whereas *R*. *porosus* was only the third (10% of shark catches)^51^. *Carcharhinus porosus* was also the most abundant shark species fished in Pará State in the early 2000s^26^ and the second most caught species in Guyana in 2015^36^. An 85% decrease of the total biomass landed in 2004 led to its classification as critically endangered in Brazil since 2014 and its harvest has been illegal ever since^16,52^. Nevertheless, the species is considered to be data deficient by IUCN, due to the lack of information from most of its range, although BNC is considered its center of abundance^52^. Data on habitat use and population genetic diversity will be essential for the development of effective conservation measures.

Other requiem sharks (*C*. *leucas*, *C*. *falciformis*, *G*. *cuvier* and *C*. *limbatus*) also identified here are all classified as Near Threatened although *C*. *falciformis* is included in Appendix II of CITES. These species comprised 11.4% of the samples, whereas they represented 16.7% of the Guyana shark fisheries^36^. Both studies had a small proportion of these large bodied species in the samples. Despite regulations, *C*. *falciformis* is one of most fished sharks worldwide, primarily as bycatch in tuna fisheries^5^. In the southern Atlantic Ocean, its population is suggested to be in decline^10^. While its harvest is not prohibited in Brazil, there are no data on catches in the BNC, and its capture by different types of fishery may be an indication of overfishing. The regular monitoring of catches in both cases will be necessary to evaluate the conservation status of this species.

One interesting finding when using both COI and NADH2 sequences was the placement of *I*. *oxyrhynchus* in the *Carcharhinus* clade together with *C*. *acronotus*, *C*. *porosus*, *C*. *limbatus*, and *C*. *leucas* (Figs 1 and 2), which is consistent with previous studies^26,42^. Given this, we suggest that a thorough taxonomic and phylogenetic revision of the genus *Carcharhinus* is performed, including *I*. *oxyrhynchus*, to test its monophyly. Perhaps this is the species that demands more immediate conservation actions since it is Critically Endangered worldwide^44,53^. Our records indicate the capture of a surprising number of specimens (n = 14) for such a depleted species. However, it was not recorded among 132 samples from Guyana^36^. This species is endemic to South America’s North Coast, thus presenting a highly restricted range^44^, and is considered to be on the brink of extinction due to its low resilience in facing the slightest fishing pressure^44^. Further studies of genetic diversity are urgently required, together with the monitoring of landings to evaluate the status of the remaining populations. Over the short term, however, harvesting should be suspended, and law enforcement strengthened by Brazilian environmental authorities and other countries included in the conservation hotspot area.

Hammerhead sharks (genus *Sphyrna*) represented almost a fifth of all samples (80 specimens, ~19%). An almost two-fold higher composition of *Sphyrna* is reported for Guyana (~37% of the identified samples), with *S*. *lewini* being the most abundant hammerhead shark in the area^36^. In the BNC, *S*. *mokarran* was the fourth most common species of all samples (9.36%) and the most common from its genus. These sharks are in extreme extinction threat worldwide, and most species are listed as Critically Endangered in Brazil. While they are not targeted specifically by fishermen, harvesting is a common practice at the BNC^39,54^. Their morphology, in particular the large body and the laterally expanded head, facilitate its capture by nets^55^. In addition, Sphyrnids are highly vulnerable to stress, and often die after capture, even if they are returned to the water alive^55^. As most of the region’s fisheries are based on trawls and gill-netting, there is considerable potential for increasingly bycatch levels for hammerheads. Our data combined with those from Kolmann *et al*.^36^ regarding hammerhead shark fishing and trading grounds suggests more effective inspections and severe law enforcement at landing and trading sites are urgent in both the BNC and Guyana to ensure the protection of such a remarkable species.

Together, other species recorded (*Mustelus canis*, *Mustelus higmani*, *Squalus* sp., and *Ginglymostoma cirratum*) corresponded to over 7% of samples. We report the genus *Mustelus* on Brazil’s North Coast for the first time using molecular data. In particular, *M*. *higmani* is an elusive species, for which few data are available. Pregnant females were recorded in the samples as bycatch of shrimp trawl fisheries. *M*. *higmani* seems to reproduce continually and thus overfishing does not seem to be a great concern for its conservation^56^, even though it composed around 40% of the shark landings in Venezuela^57^. However, the lack of data on its actual distribution in the world, abundance patterns, and the impact of trawl fisheries in its populations are important factors that need to be addressed to provide a robust conservation status conclusion.

Harvesting of *G*. *cirratum* is prohibited under Brazilian law, and its presence in the samples further reinforces the need for more effective fisheries inspections. Brazil’s North Coast represents one of the last regions of Brazil where *G*. *cirratum* captures are still common, although stocks may collapse in the near future. The reason for this potential collapse is that, even though fecundity is somewhat high with an average number of 34 embryos per litter, reproduction is biennial and a long time is necessary to reach sexual maturity^58^. Since *G*. *cirratum* is considered to be Vulnerable in Brazil and several local extirpations have occurred throughout Brazil’s coast^59^, systematic studies evaluating catch rates and size composition of captures are needed to support any conclusion on its population status in the BNC.

The first record of the genus *Squalus* for Brazil’s North Coast is intriguing, given that these sharks are thought to be restricted to deep waters^60^. Most of the 31 known *Squalus* species occur in the Indian and Pacific Oceans^60–62^, and only eight occur in the southwestern Atlantic Ocean^61–63^. The specimen was landed at Tutoia, in Maranhão state, and was probably caught in a gill net. While this may have been a random event, it shows that the deep water shark fauna of the Brazilian North Coast is poorly known. In fact, our data points that this could be a new species of *Squalus* since there was a similar match (98%) with two species that comprise a species complex just now being unraveled^60^. Comprehensive systematic surveys off the Amazon estuary and adjacent areas are urgently needed, especially given the presence of mesophotic biogenic reefs in the area^64^. In fact, the whole BNC has an enormous potential for the discovery of new species of deep water sharks, rays, and skates.

Overall, we show that threatened shark species are being harvested throughout Brazil’s North Coast, which could result in stocks collapses, local extinctions, and possibly even global extinction of some species with restricted range, like *Isogomphodon oxyrhynchus*. This prediction may be offset by the implementation of measures at a regional level to reduce shark bycatch. These could include nets with a tighter mesh attached to larger buoys, smaller soak time, and the release of live specimens. These are powerful strategies to reduce bycatch, increase the potential of releasing non-target fishes alive, and to contribute to the establishment of sustainable shark fisheries. Over the long term, more detailed research on population dynamics, genetic diversity, demographic patterns, and habitat use are crucial to subsidize the development of measures such as the establishment of priority areas for conservation. There is also an urgent need for the implementation of more effective monitoring and law enforcement for which molecular identification can be a fundamentally effective tool.

## Methods

### Sampling area

**Figure 3:**
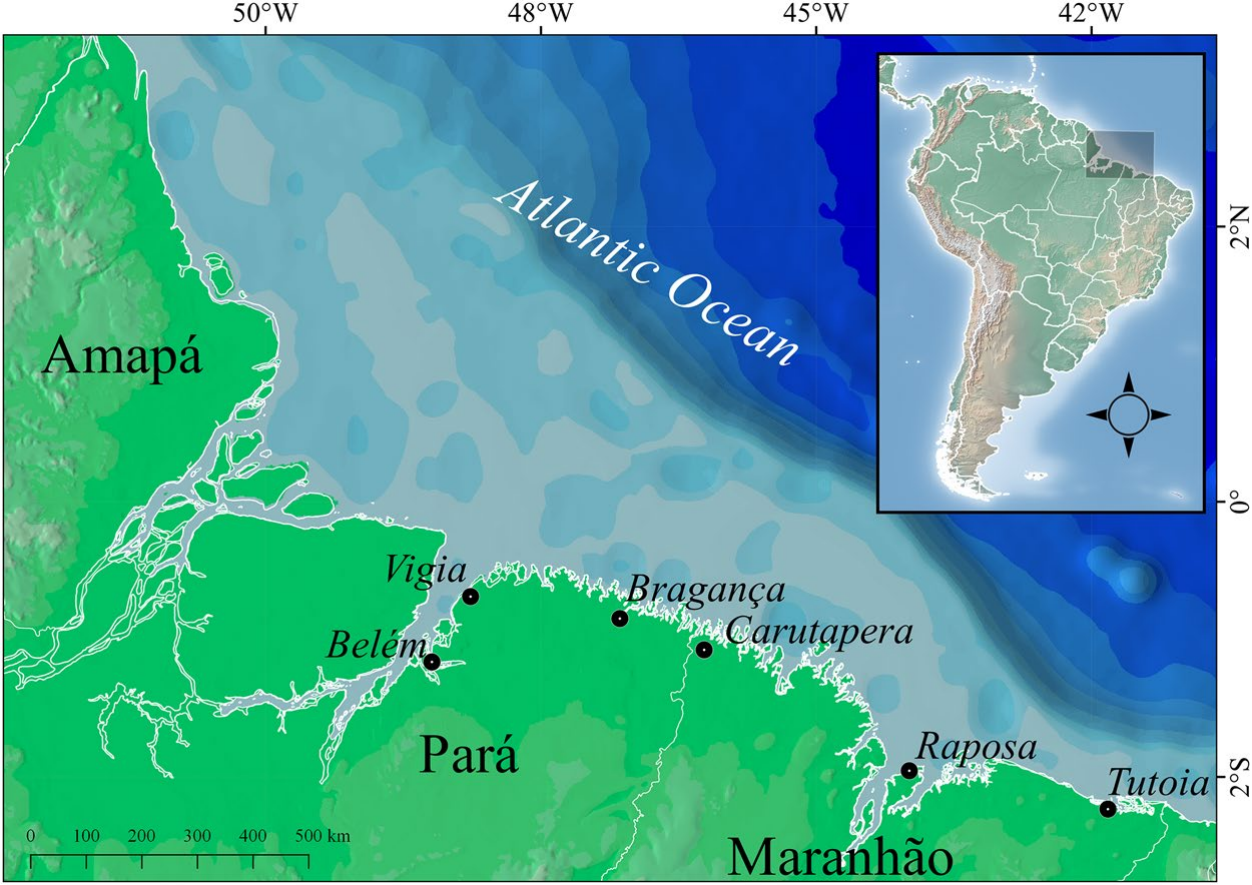
Localities on Brazil’s North Coast from which shark samples were collected. Samples from Amapá State were landed in Belém and Bragança. Map was created using QGIS version 2.18 available at www.qgis.org/en/site/.

We collected shark samples (n = 427) during both rainy and dry seasons between 2014 and 2016 from the northern Brazilian municipalities of Belém (n = 26), Bragança (n = 55), Vigia (n = 13), Carutapera (n = 14), Raposa (n = 53), and Tutoia (n = 219). These towns and cities are the most important fishery ports of Brazil’s North Coast (BNC) (Fig. 3). An additional 48 samples were obtained from CEPNOR (*Centro de Pesquisa e Gestão de Recursos Pesqueiros do Litoral Norte*), the local government fishery inspection agency. These specimens were collected off the coast of Amapá state in 2007. We acquired samples from fish markets and in artisanal fisheries landing ports (sampling permit from IBAMA/SISBIO #50091-1). Other samples were obtained as bycatch of the industrial shrimp trawlers monitored by CEPNOR in partnership with Aquatic Ecology Group from the *Universidade Federal do Pará* within the research project SHRIMP NEN (#445766/2015-8). Most of the samples (fins or muscle tissue) were from headless and finned specimens, who could not be reliably identified based solely on morphological criteria. All samples were retrieved from already fished specimens without any violations to laws regarding fisheries of endangered species.

Conservation status of each identified species was defined based on the Red List of Endangered Species of the IUCN (International Union for Conservation of Nature) and the appendices of CITES (Convention on International Trade of Endangered Species of Wild Fauna and Flora), as well as the Brazilian National List of Endangered Fauna – Fish and Invertebrates (Ordinance 445/2014, Brazilian Ministry of Environment).

### DNA-based species identification

Samples were stored in 100% ethanol and maintained at −20 °C until DNA extraction. DNA was extracted using a saline protocol^65^. Samples that did not yield DNA were extracted using the Wizard Genomics Purification kit (Promega) following the manufacturer’s Mouse Tail protocol. DNA was quantified in Nanodrop 2000 (ThermoFisher) and visualized in 1% agarose gel electrophoresis stained with GelRed (Biotium, Inc).

We used partial sequences of the mitochondrial DNA genes cytochrome oxidase subunit I (COI) and/or NADH dehydrogenase subunit 2 (NADH2) to identify species. Samples were identified using either one of the markers. The COI amplification by PCR (Polymerase Chain Reaction) used the universal FISH F1/R1 primers^41^. NADH2 was amplified using the ILEM/ASNM universal primers^42^. Reactions were carried out using 50 to 100 ηg of DNA template, 1× GoPro buffer, 1.5 mM MgCl_2_, 200 µM dNTP, 0.2 µM of each primer (COI) and 0.3 µM (NADH2), 1U of Taq polymerase (Promega), and ultrapure water to complete for 15 µL of final volume. Samples that did not yield adequate amplicons were amplified by adding 1% DMSO (dimethyl sulfoxide) to the PCR mix. Amplification conditions for COI were: denaturation with 94 °C for 2 minutes followed by 35 cycles of 94 °C for 30 seconds, 54 °C for 30 seconds, and 72 °C for 1 minute, with a final extension phase at 72 °C for 10 minutes. Conditions for NADH2 were: denaturation with 94 °C for 3 minutes followed by 39 cycles of 94 °C 30 seconds, 48 °C for 30 seconds and 72 °C for 90 seconds, and an extension phase with 72 °C for 5 minutes. Amplicons were purified using Illustra Exo Prostar (GE Healthcare Life Sciences) or PEG 8000. Sequencing was carried out using BigDye terminator v3.1 kit (Applied Biosystems) in an ABI XL 3500 (Applied Biosystems).

The quality of sequences was assessed using Geneious Pro version 9^66^. Alignments were performed using MUSCLE^67^ with the default settings. Both final alignments resulted from trimming the 5’ and 3’ ends to avoid poor base quality. Intra and inter-specific distances were calculated using Kimura-2-parameters (K2P)^68^ in MEGA 6^69^. Samples were identified using the Basic Local Alignment Research Tool (BLAST)^70^, which compared our sequences with those deposited in the Barcode of Life Database systems (BOLD) and in GenBank (National Center for Biotechnology Information - NCBI), producing similarity percentages.

Neighbor-joining trees were built for both COI and NADH2 sequences using MEGA 6 with 1000 bootstraps^71^ using the K2P distances. Sequences from *Aetobatus narinari*, *Hydrolagus africanus* and *Callorhinchus capensis* were used as outgroup for COI, and *Aetobatus narinari* and *Hydrolagus novazelandiae* for NADH2. Additional sequences of both markers from all identified species and sister-groups were retrieved from NCBI for comparison (Supplementary Table S1). Our sequences were deposited in GenBank under the following access numbers: COI: MF686569 - MF686584; NADH2: MF740888 – MF740919).

## Electronic supplementary material

Supplementary Table S1

## References

[1] Stevens JD, Bonfil R, Dulvy NK, Walker PA (2000). The effects of fishing on sharks, rays, and chimaeras (Chondrichthyans), and the implications for marine ecosystems. ICES J. Mar. Sci..

[2] Davidson LN, Dulvy NK (2017). Global marine protected areas to prevent extinctions. Nat. Ecol. Evol..

[3] Worm B (2013). Global catches, exploitation rates, and rebuilding options for sharks. Mar. Policy..

[4] Simpfendorfer CA, Dulvy NK (2017). Bright spots of sustainable shark fishing. Curr. Biol..

[5] Dent, F. & Clarke, S. *State of the global market for shark products*. FAO Fisheries and Aquaculture Technical Paper, 590 (2015).

[6] Clarke S (2008). Use of shark fin trade data to estimate historic total shark removals in the Atlantic Ocean. Aquat. Living Resour..

[7] Fabinyi M, Liu N (2014). Seafood banquets in Beijing: consumer perspectives and implications for environmental sustainability. Conservat. Soc..

[8] Jaiteh VF, Loneragan NR, Warren C (2017). The end of shark finning? Impacts of declining catches and fin demand for coastal community livelihoods. Mar. Policy.

[9] Bornatowski, H., Braga, R. R. & Barreto, R. P. Elasmobranchs consumption in Brazil: impacts and consequences. In *Advances in Marine Vertebrate Research in Latin America* (pp. 251–262). Springer, Cham (2017).

[10] Barreto R (2016). Trends in the exploitation of South Atlantic shark populations. Conserv. Biol..

[11] Barreto RR (2017). Rethinking use and trade of pelagic sharks in Brazil. Mar. Policy.

[12] Dulvy NK (2017). Challenges and Priorities in Shark and Ray Conservation. Curr. Biol..

[13] Pauly D, Zeller D (2016). Catch reconstructions reveal that global marine fisheries catches are higher than reported and declining. Nat. Commun..

[14] Reis-Filho JA, Leduc AOHC (2017). Management-Challenged Brazilian Governance and the Low Relevance of National Fishery Management Policy: Recommendations to promote viable Small-Scale Fisheries. Oceanography & Fisheries.

[15] Begossi A (2006). Temporal stability in fishing spots: conservation and co-management in Brazilian artisanal coastal fisheries. Ecol. Soc..

[16] Ministério do Meio Ambiente (MMA). Portaria MMA 125 (2014) http://www.icmbio.gov.br/portal/images/stories/docs-plano-de-acao/pan-tubaroes/portaria-125-2014-aprovacao-tubaroes.pdf (Accessed: 26th July 2017).

[17] Oliver S, Braccini M, Newman SJ, Harvey ES (2015). Global patterns in the bycatch of sharks and rays. Mar. Policy.

[18] Dulvy, N. K. *et al*. Extinction risk and conservation of the world’s sharks and rays. *Elife* e00590, 10.7554/eLife.00590.001 (2014). 10.7554/eLife.00590 PMC3897121 24448405

[19] Gemaque R (2017). Why implement measures to conserve the diversity of Elasmobranchs? The case of the northern coast of Brazil. Revista da Biologia.

[20] Clarke SC, Magnussen JE, Abercrombie DL (2006). Identification of shark species composition and proportion in the Hong Kong shark fin market based on molecular genetics and trade records. Conserv Biol..

[21] Abercrombie DL, Clarke SC, Shivji MS (2005). Global-scale genetic identification of hammerhead sharks: Application to assessment of the international fin trade and law enforcement. Conserv. Genet..

[22] Shivji MS, Chapman DD, Pikitch EK, Raymond PW (2005). Genetic profiling reveals illegal international trade in fins of the great white shark. Carcharodon carcharias. Conserv. Genet ..

[23] Liu S-YV, Chan C-LC, Lin O, Hu C-S, Chen CA (2013). DNA Barcoding of Shark Meats Identify Species Composition and CITES-Listed Species from the Markets in Taiwan. PLoS One.

[24] Chuang, P. S., Hung, T. C. & Chang, H. A. The Species and Origin of Shark Fins in Taiwan’s Fishing Ports, Markets, and Customs Detention: A DNA Barcoding Analysis. *PloS One* **11**, e0147290, 10.1371/journal.pone.0147290 (2016). 10.1371/journal.pone.0147290 PMC4723227 26799827

[25] Steinke D (2017). DNA analysis of traded shark fins and mobulid gill plates reveals a high proportion of species of conservation concern. Sci. Rep..

[26] Rodrigues-Filho LFS (2009). Identification and phylogenetic inferences on stocks of sharks affected by the fishing industry off the Northern coast of Brazil. Gen. Mol. Biol..

[27] Pinhal D (2008). Discrimination of shark species by simple PCR of 5S rDNA repeats. Genet. Mol. Biol..

[28] Mendonça FF (2010). Genetic identification of Lamniform and Carcharhiniform sharks using multiplex-PCR. Conserv Genet Resour.

[29] Pinhal D, Shivji MS, Nachtigall PG, Chapman DD, Martins C (2012). A streamlined DNA tool for global identification of heavily exploited coastal shark species (Genus *Rhizoprionodon*). PloS One.

[30] Palmeira CAM (2013). Commercialization of a critically endangered species (largetooth sawfish, *Pristis perotteti*) in fish markets of northern Brazil: authenticity by DNA analysis. Food Control.

[31] Carvalho CBV, Freitas JM (2013). The use of DNA barcoding to identify illegally traded shark fins in Brazil. Saúde, Ética & Justiça.

[32] Domingues RR, Amorim AF, Hilsdorf AWS (2013). Genetic identification of *Carcharhinus* sharks from the southwest Atlantic Ocean (Chondrichthyes: Carcharhiniformes). J. Appl. Ichthyol..

[33] Dudgeon CL (2012). A review of the application of molecular genetics for fisheries management and conservation of sharks and rays. J. Fish Biol..

[34] Hebert PDN, Cywinska A, Ball SL, de Waard JR (2003). Biological identifications through DNA barcodes. Proc. R. Soc. Lond..

[35] Sembiring A (2015). DNA barcoding reveals targeted fisheries for endangered sharks in Indonesia. Fish. Res..

[36] Kolmann MA, Elbassiouny AA, Liverpool EA, Lovejoy NR (2017). DNA barcoding reveals the diversity of sharks in Guyana coastal markets. Neotrop. Ichthyol..

[37] Henderson AC, Reeve AJ, Jabado RW, Naylor GJP (2016). Taxonomic assessment of sharks, rays and guitarfishes (Chondrichthyes: Elasmobranchii) from south‐eastern Arabia, using the NADH dehydrogenase subunit 2 (NADH2) gene. Zool. J. Linnean. Soc..

[38] Holmes BH, Steinke D, Ward RD (2009). Identification of shark and ray fins using DNA barcoding. Fish. Res..

[39] Pinhal D (2012). Cryptic hammerhead shark lineage occurrence in the Western South Atlantic revealed by DNA analysis. Mar. Biol..

[40] Ribeiro AO (2012). DNA barcodes identify marine fishes of Sao Paulo State, Brazil. Mol. Ecol. Res..

[41] Ward RD, Zemlak TS, Innes BH, Last PR, Hebert PDN (2005). DNA barcoding Australia’s fish species. Philos. Transa. R. Soc. Lond. B. Biol. Sci..

[42] Naylor, G. J. P. *et al*. Elasmobranch phylogeny: a mitochondrial estimate based on 595 species. The biology of sharks and their relatives 31–56 (2012).

[43] Menni R, Lessa RPT (1997). The Chondricthyan community off Maranhão (northern Brazil). II Biology of Species. Acta. Zool. Lilloana..

[44] Lessa R, Batista VS, Santana FM (2016). Close to extinction? The collapse of the endemic daggernose shark (*Isogomphodon oxyrhynchus*) off Brazil. Glob. Ecol. Conserv..

[45] Ward P, Myers RA (2005). Shifts in open‐ocean fish communities coinciding with the commencement of commercial fishing. Ecology.

[46] White ER, Myers MC, Flemming JM, Baum JK (2015). Shifting elasmobranch community assemblage at Cocos Island—an isolated marine protected area. Conserv. Biol..

[47] Morgan, M., Carlson, J., Kyne, P.M. & Lessa, R. 2009. *Carcharhinus acronotus*. The IUCN Red List of Threatened Species: e.T161378A5410167. Available at: 10.2305/IUCN.UK.2009-2.RLTS.T161378A5410167.en. (2009) (Accessed: 26th July 2017).

[48] Driggers WB (2004). Reproductive biology of *Carcharhinus acronotus* in the coastal waters of South Carolina. J. Fish Biol..

[49] Cortés E (2002). Incorporating uncertainty into demographic modeling: application to shark populations and their conservation. Conserv. Biol..

[50] Mattos SM, Broadhurst M, Hazin FH, Jones DM (2001). Reproductive biology of the Caribbean sharpnose shark, *Rhizoprionodon porosus*, from northern Brazil. Mar. Freshwater Res..

[51] Lessa RPT (1997). Sinopse dos estudos sobre elasmobrânquios da costa do Maranhão. Boletim do Laboratório de Hidrobiologia.

[52] Lessa, R., Almeida, Z., Santana, F. M., Siu. S. & Perez, M. *Carcharhinus porosu*s. The IUCN Red List of Threatened Species: e.T60220A12324372. Available at: 10.2305/IUCN.UK.2006.RLTS.T60220A12324372.en. (2006a). (Accessed: 26th July 2017).

[53] Lessa, R., Charvet-Almeida, P., Santana, F. M. & Almeida, Z. *Isogomphodon oxyrhynchu*s. The IUCN Red List of Threatened Species 2006: e.T60218A12323498. Available at: http://dx.doi.org/10.2305/IUCN.UK.2006.RLTS.T60218A12323498.en. (2006b). (Accessed: 26th July 2017).

[54] Almeida, Z. S. *et al*. Biodiversidade de Elasmobrânquios. In: Nunes, J. L. S. & Piorski, N. M. (Eds). *Peixes marinhos e estuarinos do Maranhão*. São Luís: Café e Lápis, pp 37–94 (2011).

[55] Gallagher AJ, Hammerschlag N, Shiffman DS, Giery ST (2014). Evolved for extinction: the cost and conservation implications of specialization in hammerhead sharks. BioScience.

[56] Tagliafico A, Hernández-Ávila I, Rangel S, Rago N (2015). Size of catch, reproduction and feeding of the small-eye smooth-hound, *Mustelus higmani* (Carcharhiniformes: Triakidae), in Margarita Island, Venezuela. Scientia Marina.

[57] Tavares R, Sánchez L, Medina E (2010). Artisanal fishery and catch structure of the smalleye smooth-hound shark, Mustelus higmani (Springer & Low 1963), from the northeastern region of Venezuela. Proc. Gulf & Carib. Fish. Inst. Cumaná, Venezuela..

[58] Castro JI (2000). The biology of the nurse shark, *Ginglymostoma cirratum*, off the Florida east coast and the Bahamas Islands. Environ. Biol. Fish..

[59] Rosa, R. S. & Gadig, O. B.F. *Ginglymostoma cirratum* (Bonnaterre, 1788). In Machado, A. B. M., Drumond, G. M. & Paglia, A. P. (eds) Livro Vermelho da Fauna Brasileira Ameaçada de Extinção, Brasília, Brasil: Ministério do Meio Ambiente, pp. 28–29 (2009).

[60] Weigmann S (2016). Annotated checklist of the living sharks, batoids and chimaeras (Chondrichthyes) of the world, with a focus on biogeographical diversity. J. Fish. Biol..

[61] Viana ST, Carvalho MR, Gomes UL (2016). Taxonomy and morphology of species of the genus *Squalus* Linnaeus, 1758 from the Southwestern Atlantic Ocean (Chondrichthyes: Squaliformes: Squalidae). Zootaxa.

[62] Viana, S. T. F. L., Lisher, M. W. & Carvalho, M. R. Two new species of short-snouted dogfish sharks of the genus *Squalu*s Linnaeus, 1758, from southernAfrica (Chondrichthyes: Squaliformes: Squalidae). *Marine Biodiversity* 1–28, 10.1007/s12526-017-0673-8 (2017).

[63] Rosa RS, Gadig OBF (2014). Conhecimento da diversidade dos Chondrichthyes Marinhos no Brasil: contribuição de José Lima de Figueiredo. Arq. Zool..

[64] Moura RL (2016). An extensive reef system at the Amazon River mouth. Sci. Adv..

[65] Aljanabi SM, Martinez I (1997). Universal and rapid salt-extraction of high quality genomic DNA for PCR-based techniques. Nucleic Acids. Res..

[66] Kearse M (2012). Geneious Basic: an integrated and extendable desktop software platform for the organization and analysis of sequence data. Bioinformatics.

[67] Edgar RC (2004). MUSCLE: multiple sequence alignment with high accuracy and high throughput. Nucleic Acids. Res..

[68] Kimura M (1980). A simple method for estimating evolutionary rates of base substitutions through comparative studies of nucleotide sequences. J. Mol. Evol..

[69] Tamura K, Stecher G, Peterson D, Filipski A, Kumar S (2013). MEGA6: Molecular Evolutionary Genetics Analysis version 6.0. Mol. Biol. Evol..

[70] Altschul SF, Gish W, Miller W, Myers EW, Lipman DJ (1990). Basic local alignment search tool. J. Mol. Biol..

[71] Felsenstein J (1985). Confidence limits on phylogenies: an approach using the bootstrap. Evol..

