# Supplementary material for: DNA-based identification reveals illegal trade of threatened shark species in a global elasmobranch conservation hotspot
Source: Sci Rep. 2018 Feb 20;8:3347. doi: 10.1038/s41598-018-21683-5 (PMC5820252; doi:10.1038/s41598-018-21683-5)
Supplement: Supplementary file 1 — Supplementary Table S1 [file 41598_2018_21683_MOESM1_ESM.pdf]

DNA-based identification reveals illegal trade of threatened shark species in a global elasmobranch conservation hotspot.

Leonardo Manir Feitosa[1]*, Ana Paula Martins[2,3], Tommaso Giarrizzo[4], Wagner Macedo[5], Iann Leonardo Monteiro[6], Romário Gemaque[6], Jorge Luiz Nunes[7], Fernanda Gomes[8], Horácio Schneider[8], Iracilda Sampaio[8], Rosália Souza[9], João Bráullio Sales[10,11], Luís Fernando Rodrigues-Filho[12], Lígia Tchaika[5], Luís Fernando Carvalho-Costa[13]

Supplementary Table S1: Access numbers for NCBI retrieved sequences used in the Neighbor-Joining trees.

Species (gene)	Access numbers
Aetobatus narinari (NADH2)	JQ518988.1
Rhizopriondon terranovae (COI)	FJ519581.1
Carcharhinus leucas (COI)	JF493063.1
Carcharhinus limbatus (COI)	GQ227282.1
Sphyrna tudes (COI)	FJ519524.1
Sphyrna mokarran (COI)	JN989316.1
Aetobatus narinari (COI)	JF492800.1
Sphyrna tiburo (COI)	KF461242.1
Rhizopriondon lalandii (COI)	ESHKD066-07
Hydrolagus novazelandiae (NADH2)	JQ518721.1
Carcharhinus porosus (COI)	ESHKC146-07
Carcharhinus acronotus (COI)	CFSAN122-11
Rhizoprionodon acutus (COI)	KF899688
Rhizoprionodon porosus (COI)	ESHKD054-07
Sphyrna lewini (COI)	KF899751
Callorhinchus capensis (COI)	JF493011.1
Hydrolagus africanus (COI)	JF493668.1
Isogomphodon oxyrhynchus(COI)	JQ518638.1
Mustelus canis (NADH2)	DQ422129.1
Sphyrna mokarran (NADH2)	DQ422103.1
Sphyrna lewini (NADH2)	JQ519063.1
Sphyrna tiburo (NADH2)	JQ518693.1
Galeocerdo cuvier (NADH2)	DQ422105.1
Carcharhinus acronotus (NADH2)	DQ422101.1
Carcharhinus limbatus (NADH2)	JN082204.1
Carcharhinus leucas (NADH2)	JQ518623.1
Carcharhinus porosus (NADH2)	JQ518614.1
Carcharhinus falciformis (NADH2)	JQ518622.1
Mustelus higmani (NADH2)	KP763717.1
Rhizoprionodon terranovae (NADH2)	JQ518651.1

Rhizoprionodon lalandii (NADH2)	JQ518646.1
Rhizoprionodon porosus (NADH2)	JQ518648.1
Sphyrna tudes (NADH2)	JQ518690.1
